# The gasdermin-D pore: Executor of pyroptotic cell death

**DOI:** 10.18632/oncotarget.11421

**Published:** 2016-09-10

**Authors:** Sebastian Rühl, Petr Broz

**Affiliations:** Focal Area Infection Biology, Biozentrum, University of Basel, Basel, Switzerland

**Keywords:** cell death, caspases

Pyroptosis is a lytic type of programmed cell death that is initiated in response to pathogen- or host-derived perturbations of the cytosol. It is characterized by cell swelling, lysis, and the release of cytoplasmic content; thus restricting the replication of intracellular pathogens and attracting effector cells of the immune system. The name pyroptosis derives from the Greek pyro (fire or fever) and ptosis (to fall), illustrating its intrinsic pro-inflammatory properties. Pyroptosis is induced by a dedicated set of proteases, the so-called inflammatory caspases, such as caspase-1, −4 and −5 in humans, and caspase-1 and −11 in mice. These caspases are activated within inflammasomes, multi-protein complexes that are assembled by cytosolic pattern-recognition receptors upon recognition various cytosolic danger- or pathogen-associated molecular patterns [1]. While the basis of inflammasome assembly and caspase activation has been well established, the exact mechanism of pyroptosis remained unclear for over a decade.

**Figure 1:**
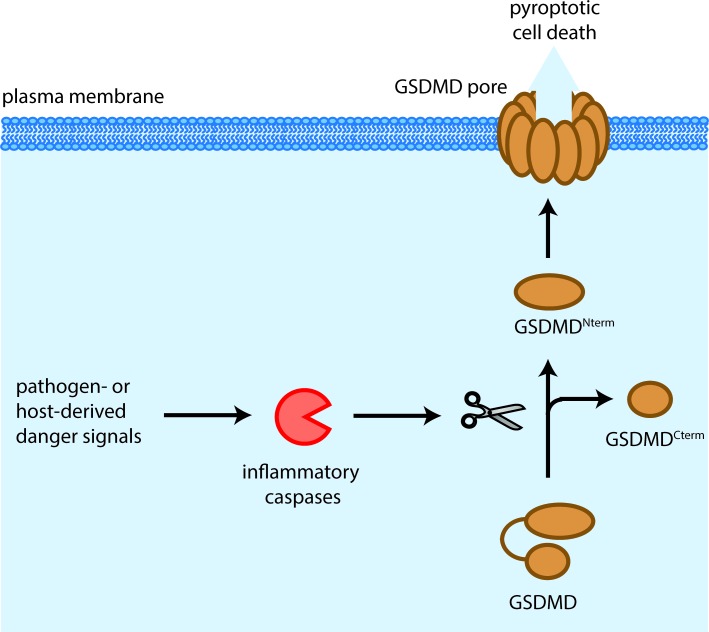
Mechanism of GSDMD-induced pyroptosis Pathogen- or host-derived signals induce the activation of inflammatory caspases, such as caspase-1 in humans and mice, caspase-4 and −5 in humans and caspase-11 in mice. These caspases cleave gasdermin D (GSDMD) resulting in the separation of the N- and C-terminal domains (GSDMD^Nterm^, GSDMD^Cterm^). The GSDMDNterm assembles large permeability pores in the plasma membrane which in a lytic type of cell death, known as pyroptosis.

This picture changed dramatically in 2015 when Shi et al. and Kayagaki et al. independently discovered that the orphan protein gasdermin D (GSDMD) was the central mediator of pyroptotic cell death downstream of both caspase-1 and caspase-11 [2, 3]. The two groups also found that GSDMD is cleaved by these caspases into a 31 kDa N-terminal fragment (GSDMD^Nterm^) and a 22 kDa C-terminal fragment (GSDMD^Cterm^), and that the N-terminus by itself had the ability to induce pyroptosis when expressed ectopically. A series of papers by us and other groups has now shown that the cytotoxicity of this N-terminal fragment is due to its ability to target, insert and permeabilize cellular membranes, therefore representing a novel class of pore forming proteins (Figure 1) [4, 5, 6, 7].

To understand how the GSDMD^Nterm^ induces pyroptosis, we started by investigating its subcellular localization using biochemical fractionation and membrane extraction methods. We found that that after caspase cleavage the cytotoxic GSDMD^N-term^ relocalized to cellular membranes and inserted into these, while the GSDMD^C-term^ remained soluble. Strikingly, the generation of the GSDMD^Nterm^ also correlated with the appearance of large plasma membrane pores and cell lysis, a characteristic feature of pyroptosis. These results indicated that GSDMD^Nterm^ either forms a pore in the host cell plasma membrane or interacts with additional proteins to promote assembly of such a pore.

To demonstrate that the GSDMD^Nterm^ is the sole executor of pyroptosis and that it has intrinsic pore forming ability, we next established a liposome-based *in vitro* assay to measure membrane permeabilization. Incubation of purified full-length GSDMD with recombinant caspase-1 in the presence of dye-loaded liposomes of different lipid compositions showed that GSDMD^Nterm^ targets membranes, whereas GSDMD^Cterm^ and full-length GSDMD remained soluble. Furthermore, this interaction resulted in a rapid release of the dye from the liposomes, indicating that the GSDMD^Nterm^ can by itself permeabilize membranes.

How does the GSDMD^Nterm^ permeabilize liposomes? To visualize the morphology of the permeabilized liposomes and possibly any pore-like structures formed by the GSDMD^Nterm^, we used a combination of Cryo-electron microscopy and atomic force microscopy (AFM). Indeed, liposomes incubated with cleaved GSDMD were covered with multiple trans-membrane holes or pores, which were as large as 20 nm in diameter, confirming our hypothesis that pyroptotic cell death is the result of the formation of a large permeability pore by GSDMD in plasma membrane of host cells.

GSDMD is just one member of the ill-characterized Gasdermin protein family that features 6 members in humans and nine in mice. Intriguingly, all gasdermin family members share similar N-terminal domains and these domains all exhibit comparable pore forming activity [2, 5]. While the exact stoichiometry and structure of the GSDMD pore has not yet been determined, Ding et al. were recently able to show that the pore formed by mouse GSDMA3 contains 16 symmetric protomers and has an inner diameter of 10-14 nm^5^. Moreover the high-resolution crystal structure of mouse gasdermin A3 revealed the structural basis for the auto-inhibitory mechanism by which the mainly α-helical C-terminus prevents the activity of the N-terminus, an architecture that is most likely conserved in the gasdermin family.

Gasdermins have emerged as a family of new class of cell death inducers, but many questions remain unanswered. For example, it is still unclear how other family members beside GSDMD are activated and how this is regulated on the post-translation level. Furthermore, if and how these proteins are implicated in physiological and pathological cell death pathways, will be an extremely interesting question to address. As caspase-11 and GSDMD were shown to be the central mediators in a murine model of sepsis, the GSDMD pore might also be a possible target for treating symptoms of septic shock. Beyond that, pyroptotic activity of all gasdermin family members could be a useful tool or target to modulate cell death across different fields of cell biology.
